# The Scholarly Journey: From Clinical Observation to Publication Excellence

**DOI:** 10.12669/pjms.42.(ICON26).15777

**Published:** 2026-04

**Authors:** Simra Naz, Rakhshanda Ayub Khan, Sayyeda Ezra Reza

**Affiliations:** 1 Ms. Simra Naz Grant Associate, Office of Research, Innovation & Commercialization (ORIC), Indus Hospital & Health Network (IHHN), Karachi Pakistan; 2 Ms. Rakhshanda Ayub Khan Research Associate, Office of Research, Innovation & Commercialization (ORIC), Indus Hospital & Health Network (IHHN), Karachi Pakistan; 3 Ms. Sayyeda Ezra Reza Manager Grants, Office of Research, Innovation & Commercialization (ORIC), Indus Hospital & Health Network (IHHN), Karachi Pakistan

The scholarly output of ICON 2026 represents the culmination of a dedicated and multifaceted editorial journey. Our theme for this cycle—”Resilient Care, Sustainable Future: Bridging Climate Action and Patient-Centered Healthcare!”—challenged our investigators to explore the vital intersection of environmental sustainability and clinical precision. To address this complex mandate, the investigative scope appeared to revolve around four core areas: the analysis of regional disease trends in Clinical Sciences and Epidemiology; the advancement of safety and empathy through Patient-Centered Care and Quality Improvement; the fortification of infrastructure via Health Systems Resilience and Digital Health; and the critical exploration of environmental impact within Climate Action and Environmental Health.^1^,^2^

Thus, the formal call for submissions initiated in May 2025, sparked a robust campaign that yielded an impressive eighty manuscripts from across the Indus Hospital and Health Network. The transition from an initial draft to a polished, journal-ready manuscript involved a rigorous process of selection, refinement, even human perseverance. This transformation took place within an energetic, time-sensitive, and academic atmosphere filled with both the excitement of exploration and the constant reality of acceptance and rejection amidst a meticulous peer review journey. We are deeply indebted to the peer reviewers whose thorough perusal facilitated this process, ensuring that a diverse and comprehensive sample of the network’s clinical experience was selected to foster an equitable dissemination of knowledge—an applaud to the effort put into each piece of writing.

This supplement is a testament to the strategic direction and guidance provided by the Chair, ICON 2026, and the unwavering support of the Secretary. Their leadership was instrumental in overcoming challenges and ensuring this supplement met the highest standards of quality and timeliness.

The final selection represents a wide cross-section of clinical excellence, comprising eighteen Original Articles, six Case Reports, and two Letters to the Editor. The unconditional acceptance of some manuscripts without a need for revisions was a reflection of the dedication and collaborative efforts involved. This speaks of our sincere appreciation for the facilitation, support, and guidance provided by the PJMS editorial team in helping us achieve this milestone once again!
